# Crystal structure of 2-*tert*-butyl-1,3-thia­zolo[4,5-*b*]pyridine

**DOI:** 10.1107/S160053681401633X

**Published:** 2014-08-01

**Authors:** Gamal A. El-Hiti, Keith Smith, Amany S. Hegazy, Ali M. Masmali, Benson M. Kariuki

**Affiliations:** a Cornea Research Chair, Department of Optometry, College of Applied Medical Sciences, King Saud University, PO Box 10219, Riyadh 11433, Saudi Arabia; b School of Chemistry, Cardiff University, Main Building, Park Place, Cardiff CF10 3AT, Wales

**Keywords:** crystal structure, C—H⋯N contacts, 1,3-thia­zolo[4,5-*b*]pyridine

## Abstract

The title compound, C_10_H_12_N_2_S, does not contain any strong hydrogen-bond donors but two long C—H⋯N contacts are observed in the crystal structure, with the most linear inter­action linking mol­ecules along [010]. The ellipsoids of the *tert*-butyl group indicate large librational motion.

## Related literature   

For the synthesis of substituted thiazolopyridines, see: Smith *et al.* (1994 ▶, 1995 ▶); El-Hiti (2003 ▶); Johnson *et al.* (2006 ▶); Rao *et al.* (2009 ▶); Sahasrabudhe *et al.* (2009 ▶); Lee *et al.* (2010 ▶); Chaban *et al.* (2013 ▶). For the crystal structure of a related compound, see: Yu * et al.* (2007 ▶).
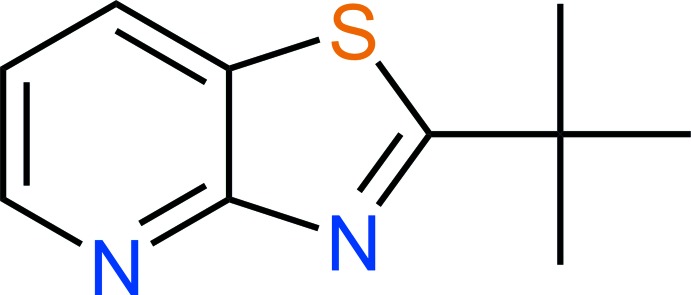


## Experimental   

### Crystal data  

C_10_H_12_N_2_S
*M* *_r_* = 192.28
Orthorhombic, 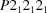

*a* = 9.4606 (3) Å
*b* = 9.7999 (3) Å
*c* = 11.1155 (4) Å
*V* = 1030.55 (6) Å^3^
*Z* = 4
Cu *K*α radiation
μ = 2.42 mm^−1^
*T* = 296 K
0.40 × 0.29 × 0.14 mm

### Data collection  

Agilent SuperNova (Dual, Cu at zero, Atlas) diffractometer
Absorption correction: multi-scan (*CrysAlis PRO*; Agilent, 2014 ▶) *T*_min_ = 0.721, *T*_max_ = 1.000
3395 measured reflections
1996 independent reflections
1951 reflections with *I* > 2σ(*I*)
*R*_int_ = 0.016

### Refinement  

*R*[*F* ^2^ > 2σ(*F* ^2^)] = 0.033
*wR*(*F* ^2^) = 0.090
*S* = 1.12
1996 reflections
121 parameters
H-atom parameters constrained
Δρ_max_ = 0.16 e Å^−3^
Δρ_min_ = −0.22 e Å^−3^
Absolute structure: Flack *x* determined using 791 quotients [(I+)-(I-)]/[(I+)+(I-)] (Parsons *et al.*, 2013 ▶).
Absolute structure parameter: 0.027 (7)

### 

Data collection: *CrysAlis PRO* (Agilent, 2014 ▶); cell refinement: *CrysAlis PRO*; data reduction: *CrysAlis PRO*; program(s) used to solve structure: *SHELXS2013* (Sheldrick, 2008 ▶); program(s) used to refine structure: *SHELXL2013* (Sheldrick, 2008 ▶); molecular graphics: *CHEMDRAW ultra* (Cambridge Soft, 2001 ▶); software used to prepare material for publication: *SHELXTL* (Sheldrick, 2008 ▶).

## Supplementary Material

Crystal structure: contains datablock(s) I, shelx. DOI: 10.1107/S160053681401633X/zs2307sup1.cif

Structure factors: contains datablock(s) I. DOI: 10.1107/S160053681401633X/zs2307Isup2.hkl

Supporting information file. DOI: 10.1107/S160053681401633X/zs2307Isup3.cml

**Fig. 1.**
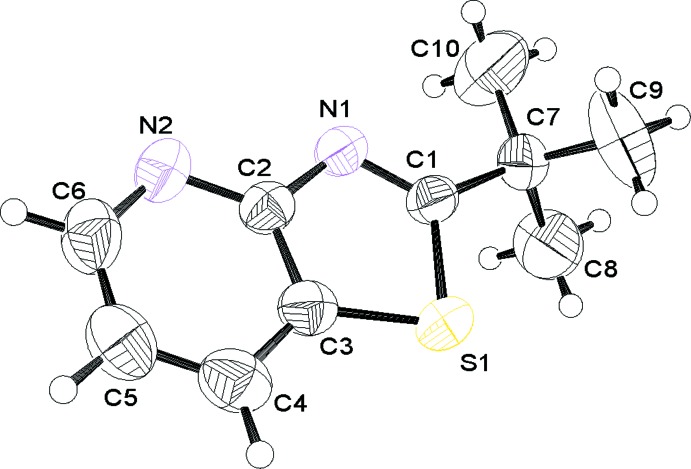
A molecule of the title compound showing atom labels and 50% probability displacement ellipsoids for non-H atoms.

. DOI: 10.1107/S160053681401633X/zs2307fig1.tif
A mol­ecule of the title compound showing atom labels and 50% probability displacement ellipsoids for non-H atoms.

**Fig. 2.**
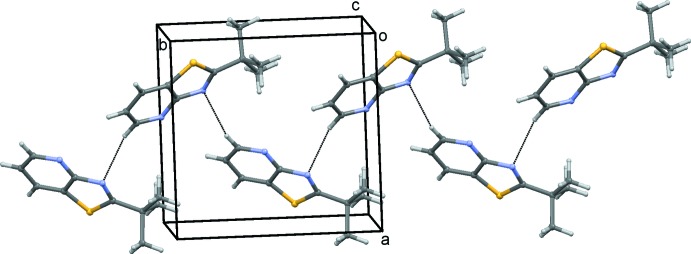
Crystal structure packing with the long linear C—H···N contacts shown as dashed lines.

. DOI: 10.1107/S160053681401633X/zs2307fig2.tif
Crystal structure packing with the long linear C—H⋯N contacts shown as dashed lines.

CCDC reference: 1013859

Additional supporting information:  crystallographic information; 3D view; checkCIF report

## Figures and Tables

**Table 1:** Hydrogen-bond geometry (Å, °)

*D*—H⋯*A*	*D*—H	H⋯*A*	*D*⋯*A*	*D*—H⋯*A*
C4—H4⋯N1^i^	0.93	2.81	3.564 (3)	138
C6—H6⋯N1^ii^	0.93	2.72	3.620 (3)	164

Symmetry codes: (i) 
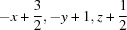
; (ii) 
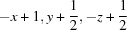
.

## supplementary crystallographic information

### S2. Structural commentary

Various methods have been reported for the synthesis of substituted thia­zolo­pyridines (Smith *et al.*, 1994, 1995; El-Hiti, 2003; Johnson *et al.*, 2006; Rao *et al.*, 2009; Sahasrabudhe *et al.*, 2009; Lee *et al.*, 2010; Chaban *et al.*, 2013). In a continuation of our research focused on new synthetic routes towards substituted heterocycles we have synthesized the title compound 2-*tert*-butyl­thia­zolo[4,5-*b*]pyridine in high yield (Smith *et al.*, 1995; El-Hiti, 2003) and now report its X-ray crystal structure. The X-ray structure for a related compound has been reported previously (Yu *et al.*, 2007). In the title compound (Fig. 1), the ellipsoids of the methyl groups of the *tert*-butyl group are large which is consistent with librational motion of the group. Assumption of a disordered model did not show significant improvement in the refinement. The molecule does not contain strong hydrogen bond donors. In the crystal, two long C—H···N contacts are observed, the most linear of which links the molecules in chains along [010] (Fig. 2).

### S5. Synthesis and crystallization

2*-tert*-Butyl­thia­zolo[4,5-*b*]pyridine was obtained in 97% yield from acid hydrolysis of 3-(diiso­propyl­amino­thio­carbonyl­thio)-2-(pivalamido)­pyridine under reflux (Smith *et al.*, 1995). The compound may also be synthesized in 66% yield from reaction of 3-(diiso­propyl­amino­thio­carbonyl­thio)-2-amino­pyridine with 2,2-di­methyl­propionic acid in the presence of phospho­rus oxychloride under reflux (El-Hiti, 2003). Crystallization from a mixture of ethyl acetate and di­ethyl ether (1:3 by volume) gave the title compound as colourless crystals. The NMR and low and high resolution mass spectra for the title compound were consistent with those previously reported (Smith *et al.*, 1995).

### S6. Refinement

Crystal data, data collection and structure refinement details are summarized in the crystal data table. The hydrogen atoms were positioned geometrically and refined using a riding model with *U*_iso_(H) = 1.2 times *U*_eq_ for the atom to which they are bonded in the case of aromatic rings, NH and CH_2_ groups and 1.5 times *U*_eq_ for the methyl hydrogens. Crystal data, data collection and structure refinement details are summarized in Table 1. The Flack parameter (Parsons *et al.*, 2013) was 0.027 (7) but is not of any structural relevance with this compound.

### Crystal data

**Table:**

C_10_H_12_N_2_S	*D*_x_ = 1.239 Mg m^−^^3^
*M**_r_* = 192.28	Cu *K*α radiation, λ = 1.54184 Å
Orthorhombic, *P*2_1_2_1_2_1_	Cell parameters from 1951 reflections
*a* = 9.4606 (3) Å	θ = 6.0–73.4°
*b* = 9.7999 (3) Å	µ = 2.42 mm^−^^1^
*c* = 11.1155 (4) Å	*T* = 296 K
*V* = 1030.55 (6) Å^3^	Plate, colourless
*Z* = 4	0.40 × 0.29 × 0.14 mm
*F*(000) = 408	

### Data collection

**Table:**

Agilent SuperNova (Dual, Cu at zero, Atlas) diffractometer	1951 reflections with *I* > 2σ(*I*)
Radiation source: SuperNova (Cu) X-ray Source	*R*_int_ = 0.016
ω scans	θ_max_ = 73.4°, θ_min_ = 6.0°
Absorption correction: multi-scan (*CrysAlis PRO*; Agilent, 2014)	*h* = −7→11
*T*_min_ = 0.721, *T*_max_ = 1.000	*k* = −11→12
3395 measured reflections	*l* = −13→10
1996 independent reflections	

### Refinement

**Table:**

Refinement on *F*^2^	Hydrogen site location: inferred from neighbouring sites
Least-squares matrix: full	H-atom parameters constrained
*R*[*F*^2^ > 2σ(*F*^2^)] = 0.033	*w* = 1/[σ^2^(*F*_o_^2^) + (0.0513*P*)^2^ + 0.0815*P*] where *P* = (*F*_o_^2^ + 2*F*_c_^2^)/3
*wR*(*F*^2^) = 0.090	(Δ/σ)_max_ < 0.001
*S* = 1.12	Δρ_max_ = 0.16 e Å^−^^3^
1996 reflections	Δρ_min_ = −0.22 e Å^−^^3^
121 parameters	Absolute structure: Flack x determined using 791 quotients [(I+)-(I-)]/[(I+)+(I-)] (Parsons *et al.*, 2013).
0 restraints	Absolute structure parameter: 0.027 (7)

### Special details

**Table:**

Geometry. All e.s.d.'s (except the e.s.d. in the dihedral angle between two l.s. planes) are estimated using the full covariance matrix. The cell e.s.d.'s are taken into account individually in the estimation of e.s.d.'s in distances, angles and torsion angles; correlations between e.s.d.'s in cell parameters are only used when they are defined by crystal symmetry. An approximate (isotropic) treatment of cell e.s.d.'s is used for estimating e.s.d.'s involving l.s. planes.

### Fractional atomic coordinates and isotropic or equivalent isotropic displacement parameters (Å^2^)

**Table:**

	*x*	*y*	*z*	*U*_iso_*/*U*_eq_	
C1	0.8245 (2)	0.2846 (2)	0.37042 (19)	0.0458 (5)	
C2	0.6967 (2)	0.4685 (2)	0.33748 (19)	0.0435 (5)	
C3	0.7716 (2)	0.5154 (3)	0.4378 (2)	0.0483 (5)	
C4	0.7455 (3)	0.6454 (3)	0.4822 (3)	0.0645 (6)	
H4	0.7924	0.6789	0.5496	0.077*	
C5	0.6470 (3)	0.7218 (3)	0.4218 (3)	0.0677 (7)	
H5	0.6260	0.8097	0.4477	0.081*	
C6	0.5791 (3)	0.6682 (3)	0.3225 (3)	0.0656 (7)	
H6	0.5138	0.7234	0.2833	0.079*	
C7	0.8831 (3)	0.1417 (2)	0.3577 (2)	0.0556 (6)	
C8	0.8599 (5)	0.0641 (4)	0.4747 (3)	0.0938 (11)	
H8A	0.9096	0.1090	0.5387	0.141*	
H8B	0.8948	−0.0274	0.4662	0.141*	
H8C	0.7608	0.0617	0.4929	0.141*	
C9	1.0411 (4)	0.1506 (5)	0.3319 (5)	0.1151 (16)	
H9A	1.0560	0.1983	0.2575	0.173*	
H9B	1.0798	0.0603	0.3260	0.173*	
H9C	1.0871	0.1990	0.3961	0.173*	
C10	0.8067 (6)	0.0657 (4)	0.2577 (4)	0.124 (2)	
H10A	0.7076	0.0614	0.2758	0.186*	
H10B	0.8440	−0.0251	0.2513	0.186*	
H10C	0.8203	0.1129	0.1829	0.186*	
N1	0.72864 (19)	0.3364 (2)	0.30173 (16)	0.0453 (4)	
N2	0.6004 (2)	0.5424 (2)	0.2787 (2)	0.0576 (5)	
S1	0.88585 (7)	0.39028 (7)	0.48644 (5)	0.0607 (2)	

### Atomic displacement parameters (Å^2^)

**Table:**

	*U*^11^	*U*^22^	*U*^33^	*U*^12^	*U*^13^	*U*^23^
C1	0.0431 (10)	0.0571 (12)	0.0371 (10)	0.0007 (10)	−0.0013 (8)	0.0016 (9)
C2	0.0423 (10)	0.0483 (11)	0.0398 (10)	−0.0043 (9)	0.0022 (8)	0.0053 (9)
C3	0.0443 (11)	0.0550 (12)	0.0457 (11)	−0.0048 (9)	0.0022 (9)	−0.0035 (9)
C4	0.0664 (15)	0.0611 (14)	0.0661 (15)	−0.0075 (11)	0.0034 (13)	−0.0160 (13)
C5	0.0707 (17)	0.0500 (13)	0.0823 (19)	−0.0008 (12)	0.0129 (15)	−0.0023 (13)
C6	0.0686 (16)	0.0542 (13)	0.0740 (17)	0.0102 (12)	0.0041 (14)	0.0157 (13)
C7	0.0564 (12)	0.0560 (13)	0.0544 (12)	0.0118 (11)	−0.0010 (11)	0.0012 (10)
C8	0.122 (3)	0.078 (2)	0.081 (2)	0.0248 (19)	0.009 (2)	0.0219 (17)
C9	0.074 (2)	0.097 (3)	0.174 (5)	0.0241 (19)	0.044 (3)	−0.007 (3)
C10	0.187 (5)	0.073 (2)	0.112 (3)	0.052 (3)	−0.072 (3)	−0.034 (2)
N1	0.0468 (9)	0.0492 (9)	0.0401 (9)	0.0001 (8)	−0.0045 (8)	0.0005 (8)
N2	0.0588 (11)	0.0570 (11)	0.0570 (12)	0.0070 (10)	−0.0078 (10)	0.0101 (9)
S1	0.0568 (3)	0.0761 (4)	0.0493 (3)	0.0096 (3)	−0.0161 (3)	−0.0112 (3)

### Geometric parameters (Å, º)

**Table:**

C1—N1	1.290 (3)	C6—H6	0.9300
C1—C7	1.512 (3)	C7—C10	1.521 (4)
C1—S1	1.753 (2)	C7—C8	1.522 (4)
C2—N2	1.335 (3)	C7—C9	1.524 (4)
C2—N1	1.387 (3)	C8—H8A	0.9600
C2—C3	1.399 (3)	C8—H8B	0.9600
C3—C4	1.389 (4)	C8—H8C	0.9600
C3—S1	1.722 (3)	C9—H9A	0.9600
C4—C5	1.371 (4)	C9—H9B	0.9600
C4—H4	0.9300	C9—H9C	0.9600
C5—C6	1.380 (4)	C10—H10A	0.9600
C5—H5	0.9300	C10—H10B	0.9600
C6—N2	1.341 (4)	C10—H10C	0.9600
			
N1—C1—C7	124.6 (2)	C8—C7—C9	109.3 (3)
N1—C1—S1	115.80 (18)	C7—C8—H8A	109.5
C7—C1—S1	119.64 (17)	C7—C8—H8B	109.5
N2—C2—N1	121.0 (2)	H8A—C8—H8B	109.5
N2—C2—C3	123.9 (2)	C7—C8—H8C	109.5
N1—C2—C3	115.1 (2)	H8A—C8—H8C	109.5
C4—C3—C2	119.7 (2)	H8B—C8—H8C	109.5
C4—C3—S1	130.8 (2)	C7—C9—H9A	109.5
C2—C3—S1	109.55 (18)	C7—C9—H9B	109.5
C5—C4—C3	116.6 (3)	H9A—C9—H9B	109.5
C5—C4—H4	121.7	C7—C9—H9C	109.5
C3—C4—H4	121.7	H9A—C9—H9C	109.5
C4—C5—C6	120.0 (3)	H9B—C9—H9C	109.5
C4—C5—H5	120.0	C7—C10—H10A	109.5
C6—C5—H5	120.0	C7—C10—H10B	109.5
N2—C6—C5	124.8 (3)	H10A—C10—H10B	109.5
N2—C6—H6	117.6	C7—C10—H10C	109.5
C5—C6—H6	117.6	H10A—C10—H10C	109.5
C1—C7—C10	110.3 (2)	H10B—C10—H10C	109.5
C1—C7—C8	109.3 (2)	C1—N1—C2	110.6 (2)
C10—C7—C8	108.1 (3)	C2—N2—C6	115.0 (2)
C1—C7—C9	108.9 (3)	C3—S1—C1	88.96 (11)
C10—C7—C9	110.9 (3)		

### Hydrogen-bond geometry (Å, º)

**Table:**

*D*—H···*A*	*D*—H	H···*A*	*D*···*A*	*D*—H···*A*
C4—H4···N1^i^	0.93	2.81	3.564 (3)	138
C6—H6···N1^ii^	0.93	2.72	3.620 (3)	164

Symmetry codes: (i) −*x*+3/2, −*y*+1, *z*+1/2; (ii) −*x*+1, *y*+1/2, −*z*+1/2.
