# Supplementary figures and images for: Crystal structure of 2-tert-butyl-1,3-thia­zolo[4,5-b]pyridine
Source: Acta Crystallogr Sect E Struct Rep Online. 2014 Aug 1;70(Pt 9):o932. doi: 10.1107/S160053681401633X (PMC4186112; doi:10.1107/S160053681401633X)

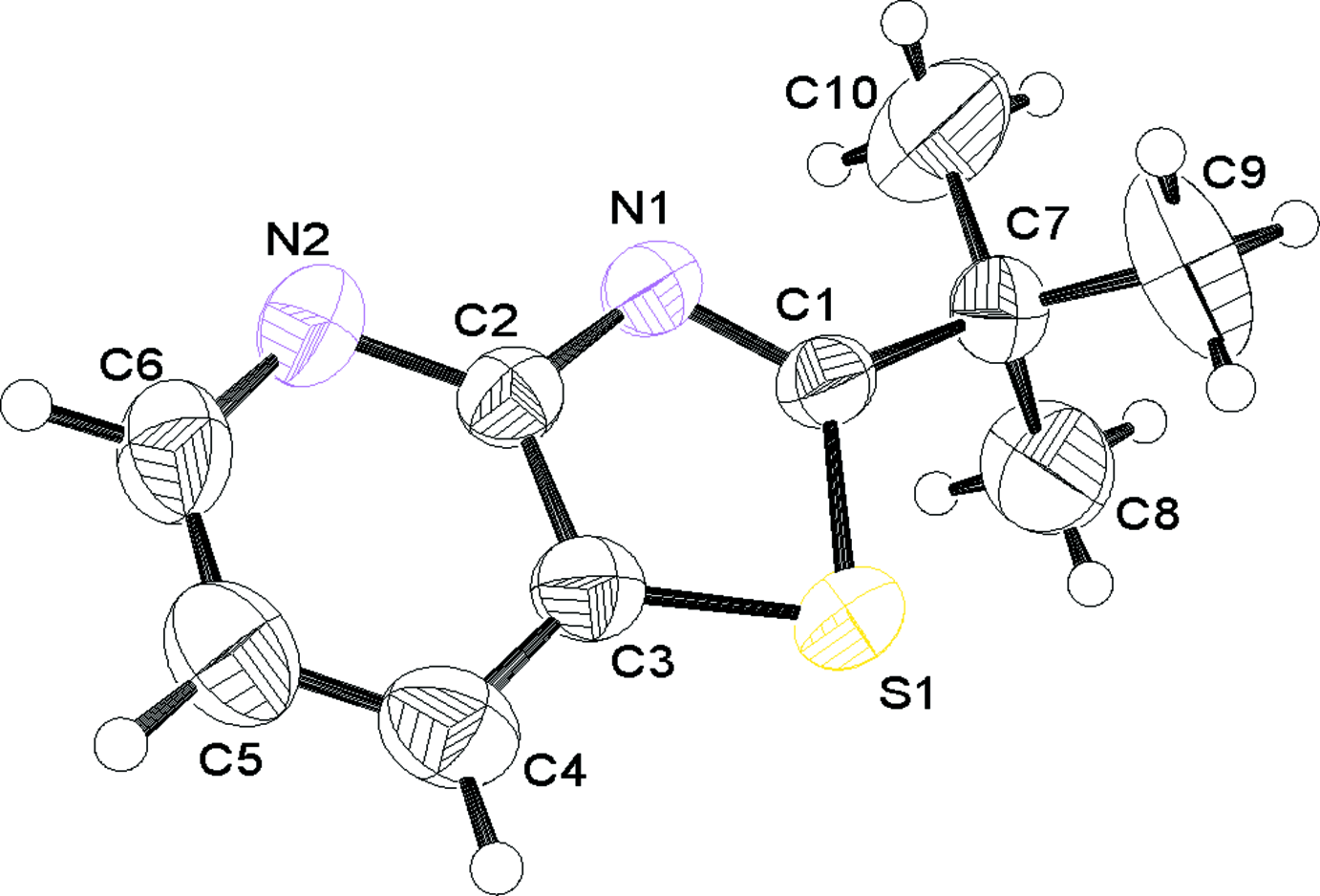

Supplement: Supplementary file 4 [file e-70-0o932-fig1.tif]

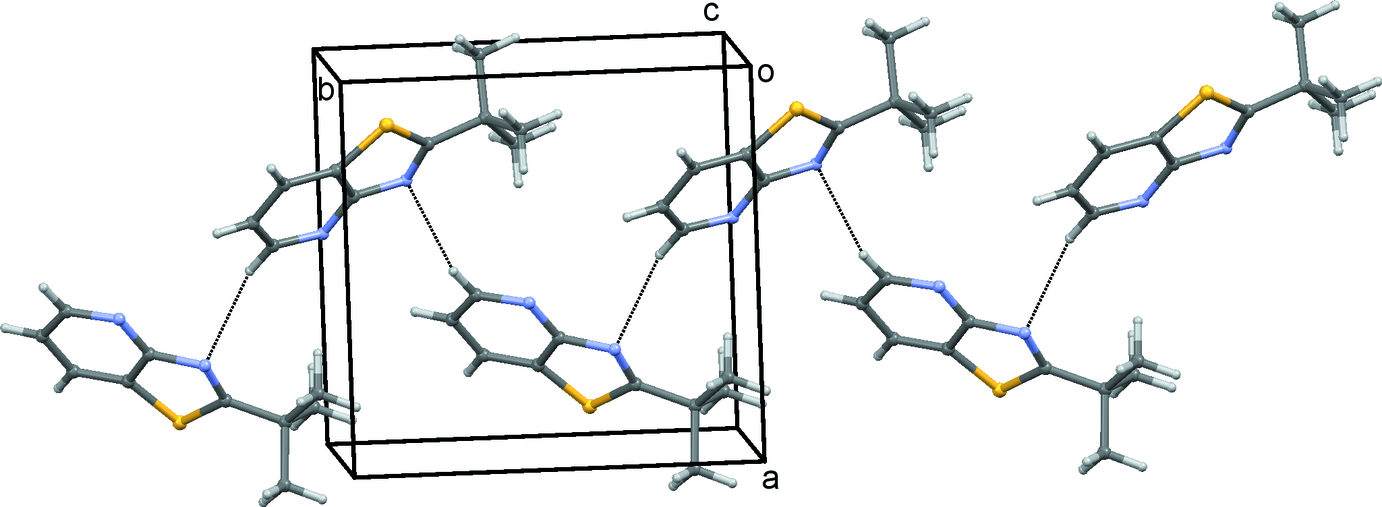

Supplement: Supplementary file 5 [file e-70-0o932-fig2.tif]
